# Observations on Functional Disorders of the Spinal Cord, and Their Connexion with Hysterical, Nervous, and Other Diseases; Illustrated by Cases, Selected Chiefly from the Reports of the Pallas- Kenry and Currah Dispensaries

**Published:** 1830-11

**Authors:** Wm. Griffin, D. Griffin

**Affiliations:** Limerick.; Limerick.


### FUNCTIONAL DISORDERS OF THE SPINAL CORD.

*Observations on Functional Disorders of the Spinal Cord, and their Connexion with Hysterical, Nervous, and other Diseases; illustrated by Cases, selected chiefly from the Reports of the Pallas-Kenry and Currah Dispensaries.* By WM. GRIFFIN, M.D. and D. GRIFFIN, Esq. M.R.C.S. Limerick.

(Continued from vol. liii. p. 389.)

AFFECTIONS of the stomach, as sickness, vomiting, pain, &c. dependent on irritation at the origin of the eighth pair, are exceedingly common; and it is of the greatest importance in practice to have them fully understood. Notwithstanding the particular investigations of many eminent physiologists, our knowledge of the functions of these nerves is still so very uncertain, that our opinions on the subject must be formed principally by the consideration of such pathological facts as come within our experience. Cases have been already related in which nausea and vomiting were constant and uncontrollable symptoms, coexistent with tenderness of the cervical spine, and whose whole progress could leave little rational doubt as to their dependence: but we shall presently offer others of a still less disputable character, and which must at the least prove that the pneumogastric is a nerve of peculiar, if not of common sensation. The supposition that it does possess even common sensation seems to be supported by the strongest probabilities, although in a late publication, of which we have before spoken, an opposite opinion is maintained.* As the experiments put forward in proof of that opinion appear at a cursory glance very conclusive, it is necessary to show that others, equally satisfactory, favor our own conjecture, before offering any reason suggested merely by the observable phenomena of disease.

With regard to its being a motor nerve, "the analogy," it is observed in the work alluded to, "between the portio dura of the seventh and the pneumogastric, is so striking as to excite the strongest suspicions that their functions or properties are similar: they both arise by a single series of fibrils from a distinct portion of the spinal marrow, and not by double origins, or distinct roots from the two great spinal columns, like the fifth pair and spinal nerves."

Now it would appear to us, that the analogy between the fifth and eighth is infinitely more striking; the former supplying or becoming accessory to the sensations of the organs of the intellect and of the peculiar senses; the latter of the organs of respiration and digestion: and in this view these two nerves certainly become the most important and interesting of any in the whole frame. There would seem to be some error in the anatomical statement of the origin of the pneumogastric, as a small ganglia, the sign of its connexion with the posterior column, is formed both on the glosso-pharyngeal and itself; and hence MAGENDIE, BELL, MAYO, and WILSON PHILIP, all unhesitatingly assert that they possess sensibility. It is to be remembered also that the muscular fibres of the stomach cannot be excited by irritating the eighth pair, in the way a muscle of voluntary motion may, and that, as Magendie has shown, even the gizzards of birds will not contract by such irritation; a fact totally discordant with the supposed analogy between it and the seventh: and it fails yet further in its being apparently superfluous as a mere motor nerve, since the motions of the stomach and intestines are quite independent of it. This is not at all the case with the seventh and the parts it supplies, nor even with that portion of the eighth which is acknowledged on all hands to possess a motor power; those branches which supply the dilators of the glottis, and preside over the alternate contraction and relaxation of the lower third of the œsophagus.

It may be inquired, if the eighth pair be simply motor nerves, what supplies the stomach with sensation when the

* "Nothing further, I presume, need be said to prove that the pneumogastric is not a nerve of sensation, and consequently that it cannot be the seat of pain."
TEALE *on Neuralgic Diseases.*

spinal nerves are palsied and insensible. In the case of Palmer, related by Mr. C. Bell, the abdominal muscles hung loose and inactive, and the skin senseless, in consequence of disease of the cervical vertebræ, but the stomach retained its sensibility; it is fair, he says, to conclude through the influence of the par vagum.

Again, if there is any fact in pathology at all clear, we think it is that irritation may be propagated by this nerve, from its capillary extremities to its origin in the brain, and it appears as impossible to us that a mere motor nerve should transmit this irritation, as that it should impart to us the consciousness or intelligence of its own actions. This it is not easy to conceive it does, as it seems impossible for two qualities or powers, however subtle, to traverse the same medium at the same moment in opposite currents; a volition passing out and a sensation returning.

But there are many more positive reasons for our attributing to it at least a peculiar sensibility. Dr. Wilson Philip, whose inquiries have been so specially directed to its uses, and who has given the subject a rare attention, believes that the peculiar sensation obliging respiration belongs to it. "The part of the brain," he observes, "to which impressions from the lungs are conveyed is evidently that, where the eighth pair of nerves which supplies them originates, and from which the spinal marrow proceeds. These nerves are nowise connected with the muscles of respiration, they only convey the sensations excited in the lungs; yet it appears from the experiments in which M. Le Gallois removed the brain by slices, that respiration continued until he removed this part of it, and then instantly ceased. In these experiments the power of the muscles of respiration, and the nervous power which excites them, still remain, as may be easily ascertained by stimulants properly applied to the spinal marrow. It is the sensation which excites to inspire, that is, the influence of the sensorial power which is withdrawn." In full accordance with this, we may mention Magendie's statement, that the action of the posterior portion of the fourth ventricle is in strict harmony with that of the eighth pair; his suggestion that appearances of disease may be found there in asthmatic cases; and our own frequent experience of oppressions connected with tenderness at the upper cervical vertebræ. It would seem equally probable that the peculiar sensations of the stomach depend on this nerve. Nausea and attempts at vomiting may occur after division of the eighth pair, and even after excision of the stomach, because the sensations reside in the posterior portion of the fourth ventricle; but if this was disorganised, no such sensations, we believe, could be experienced. Magendie has shown that vomiting from an emetic may be repressed by compressing the fourth ventricle at the origin of the par vagum; and we shall find many cases in which uncontrollable sickness and retching was at once allayed by applying a blister to the upper cervical vertebræ. Instances of spasm of stomach terminating fatally are met with, in which, on examination, no diseased appearances could be detected, except at the base of the brain; and we may further refer to the frequent occurrence of a total disrelish of food, or of greedy hunger, in certain cerebral affections.

Desmoulin and Magendie, in their late work, after detailing numerous experiments illustrative of some of the uses of this nerve, conclude that it most probably serves to establish intimate relations between the stomach and the brain, to transmit impressions giving notice whether any noxious substance has entered, and whether it is capable of being digested.

It only remains to make a few observations on Mr. Broughton's experiments, which, however apparently absolute, by no means seem so on examination. In the first place, the principle can scarcely be universally admitted that, on pricking a sensitive nerve, the animal must necessarily complain of pain: in fact, in the very experiments quoted, in which the par vagum was pierced by a pin, pinched, and slowly cut through with a scissors, pain should have been experienced, at all events, at the larynx, pharynx, and œsophagus; as all our best physiologists seem to agree that the fibrils of that nerve distributed to these parts at all events are sensitive, as well as motory. It should have been considered, too, that the sympathetic, to establish whose sensibility this property is denied to the par vagum, is itself totally devoid of sensation, and may be cut and mangled and removed to an incalculable extent, as Magendie asserts, not only without pain, but without manifest disorder of any kind.

It should be still further considered that, although pain may be occasioned by pinching or injuring a nerve of common sensation, the instrument of a peculiar sense cannot be called into action by any but its own appropriate irritation. Pinching the origin of the optic or olfactory nerve could never occasion vision or hearing; the pneumogastric may be a nerve, therefore, of a peculiar, if not of a common sensibility.

But when we reflect on all that has been said, its strong analogy with the fifth, which is a motor nerve to one set of organs, a nerve of common sensation to another, and a nerve of peculiar sensation to a third; when we reflect that pain of stomach is a still more common attendant on cerebral disease than hunger or nausea; that it is met with in nearly all cases of cervical tenderness, and that the gastric soreness in typhus is almost invariably accompanied by a similar symptom; it will not be thought too much to infer a strong probability that this nerve possesses even common sensation.

We now proceed to show that these several affections, sickness and vomiting, hunger and loss of appetite, pain or tenderness of stomach, which are generally, both in practice and in the schools, attributed to some diseased or disordered state of the organ itself are very frequently dependent on disturbance at the origin of the par vagum, and always in those instances indicated by tenderness at the cervical portion of the spine.

We shall first relate a case proving the dependence of vomiting on injury of the cervical spine, although very unsatisfactory with respect to any inference deducible from the treatment.

XXXIII. Michael Guerin, aged sixteen years, was struck with a hurly on the back of the neck, about the situation of the fourth cervical vertebra. The blow was so violent that it brought him to the ground. He was removed to bed, and was soon after seized with vomiting: he complained of *tenderness of stomach*, had a hard, quick, irregular pulse, with whiteness of tongue and thirst, but no pain. There was no mark where the hurly had struck him, though the part was sore to the touch. He was bled and purged, and the neck repeatedly blistered within a few days; he had effervescent draughts, opiates, and other remedies, and all with very little relief to the symptoms, except that the thirst and feverishness subsided, and the pulse became somewhat softer and slower. A degree of subsultus tendinum, which had shown itself on the second or third day after the accident, was also diminishing, and eventually wore away. He now lay in the bed with a cool skin, a natural countenance, the tongue almost clear, and the spirits undepressed; yet he was unable to retain the smallest particle of food or drink for one moment on his stomach. After four or five weeks passed in this manner, during which nothing seemed so remarkable as the little emaciation or debility induced, notwithstanding the continual rejection of the food, a gradual improvement took place, and the stomach eventually became tranquil, and recovered its tone.

There appeared to be little or no nausea in the foregoing case, and the food was ejected without force or distress, as in one or two instances mentioned by Dr. Brown: this is, however unusual, and chiefly observable in protracted attacks. The following instance may serve to illustrate this; but it will be found chiefly interesting from the resemblance it bears to a severe inflammatory attack, and the obvious correctness of the diagnosis furnished by the spinal tenderness.

XXXIV. A lady, aged fifty-six, was seized with violent pain in the pit of the stomach and right side, attended with excessive soreness along the margin of the lower ribs back to the spine: there was constant distressing nausea and vomiting, heat of skin and thirst, quickness of pulse, and furred tongue; the pain came on at intervals with increased violence, like paroxysms of colic; and, after some hours, the tenderness of stomach and side became so acute that the weight of the bedclothes could scarce be borne. In detailing all the symptoms with which she was affected, and speaking of her headach, she happened to mention that she felt a stiffness of the muscles of her jaws, as if she was about to be attacked with locked jaw. This immediately suggested an examination of the spine; and extreme tenderness of the lower cervical vertebræ, of all the dorsal from the fourth downwards, and of the superior lumbar, was discovered, although she did not herself previously suspect its existence. There is, perhaps, generally no objection to the abstraction of a small quantity of blood in these severe cases, even in the belief, as in this attack, that they are not inflammatory; but from the general feebleness of the lady's constitution, it was deemed unnecessary. The vomiting, which had continued for two days previous to her sending for assistance, and resisted other usual remedies, was relieved by a blister to the stomach, where it was applied rather than to the nape of the neck, at the anxious desire of the patient. Irritation at the trunk of a nerve is sometimes unaccountably relieved by blistering the minute extremities, as Mr. Brodie has shown in the occasional benefit derived from blistering the knee in affections of the hip-joint, and, as it is frequently successful in allaying irritability of stomach, from whatever cause it arises, when directly applied, it was not thought necessary, in opposition to the lady's wishes, to make the application now to an unusual place, where the advantage could be considered only as conjectural. Her recovery, which was frequently interrupted by a recurrence of the vomiting, though not to so great a degree, proved very slow, and was effected chiefly by the use of continued mild purgatives, with anodynes, and a strict attention to diet.

In some weeks after her apparent recovery, however, the vomiting returned, with its concomitant, the tenderness of the cervical vertebræ, but without any serious illness or distressing symptoms. It was invariably brought on by taking food; but there was now no nausea and no feeling of distress, the contents of the stomach were simply rejected. She eventually gave up eating breakfast altogether, and confined herself to one meal a day (her dinner), which, however, she never retained long. After several weeks had been passed in this way, it was singular to observe, as in the case of the boy, how little there seemed to be of debility or emaciation; there was scarcely any change in the lady's appearance. As she had similar attacks before, at different periods of her life, with long intervals of tolerable health, it seemed fair to infer that the present was not connected with organic disease. Attention to the general health, and to the cervical portion of the spine, were therefore recommended. Whether this was observed, or how much longer the affection lasted, we have not learned.

The beneficial effect of applications to the cervical portions of the spine, in allaying nausea and vomiting, which were in the foregoing case only conjectured, we had afterwards many opportunities of witnessing in the more complex instances of spinal irritation. The following seems particularly illustrative, although not exactly of that class.

XXXV. In an accidental visit to the House of Industry, a patient was pointed out, who had not kept any thing on her stomach for five months: she was a young girl, about twenty-five years of age, and was pale, weak, and greatly emaciated; she was constantly confined to bed; the catamenia was suppressed for seven months; she had taken much medicine without any benefit. On examination there was found extreme tenderness of the second, third, and fourth cervical vertebræ; the slightest touch making her shrink and gather her features; there was no soreness or hardness in the hepatic or gastric regions, but the abdomen was much sunk, as is usual where there is general wasting. From the appearance of the patient, little was expected from any remedy, except perhaps some alleviation of suffering; and, as the vomiting was one of the most distressing symptoms, a blister was ordered to be applied to the tender cervical vertebræ. In a few days afterwards we learnt that, as soon as the blister had risen well, the vomiting immediately ceased, and did not seem inclined to recur. Directions were, however, given to repeat the application, which was accordingly done, and, after the lapse of several days more, there was no return of the complaint: she was perfectly free from vomiting, and entertained hopes of recovery.

We had no opportunity of witnessing the progress of this case, but heard that in some time she grew very ill again, and at last died. There was no inspection of the body. It was a singular circumstance that her bowels had been for a long period very costive, so as scarcely to be moved by any medicine; but after the application of the blister, they became perfectly free, and even evinced a disposition to diarrhœa.

XXXVI. A young lady, fair, and of delicate make, felt a gradual and uncontroulable langour gaining upon her, attended by frequent headach and loss of appetite. After continuing in this state for some weeks, she was attacked with sickness of stomach and violent ineffectual efforts at vomiting, brought on by the slightest twisting or bending of the frame, or raising herself to the erect position. It was accompanied by swimming of the head and giddiness, with distressing headach, and followed by frequent fits of syncope, or insensibility, in which she used to remain for one or two hours, pale, cold, and apparently lifeless. She was sometimes affected with palpitations and great sense of sinking, with pain in the stomach and sides, especially the right, and extreme coldness of the feet; she had no appetite; the pulse was eighty; the skin cooler than natural; the tongue clean. The pain, sickness of stomach, and retching, were considerably relieved as long as she remained in the horizontal position, but the slightest motion of the trunk occasioned their instant recurrence, so that, when making the bed, it was found necessary to remove her in a recumbent position. She constantly complained of inexpressible languor. There was acute tenderness at the first and second cervical vertebræ, and it existed in a less degree in all the dorsal.

She was for some days treated by saline draughts, opiates, antispasmodics, and mild purgatives, but with little apparent benefit: the headach, giddiness, retching, and fits of insensibility, still continuing to recur on the slightest movement. A blister to the neck was then ordered, and there was immediate relief to all the symptoms: even the pain in the sides abated, and warmth returned to the skin. We could not learn that there had been any suppression or interruption of the catamenia; but they occurred on the next night, and the young lady was up and well in a few days.

(To be continued.) Vol. lxvi. p. 1.

V

